# Mortality and COVID Infection: Predictors of Mortality 10 Months after Discharge

**DOI:** 10.3390/diseases12060123

**Published:** 2024-06-05

**Authors:** Víctor Vera-Delgado, Dácil García-Rosado, Onán Pérez-Hernández, Esther Martín-Ponce, Alejandro Mario de La Paz-Estrello, Cristina García-Marichal, Sergio Pérez-Fernández, Valle Rodríguez-Morón, Remedios Alemán-Valls, Emilio González-Reimers, Candelaria Martín-González

**Affiliations:** 1 Servicio de Medicina Interna, Hospital Universitario de Canarias, 38320 San Cristóbal de La Laguna, Spain; victoringui@gmail.com (V.V.-D.); dacilgarsado@gmail.com (D.G.-R.); onanperezhernandez@gmail.com (O.P.-H.); esthermartinp@yahoo.es (E.M.-P.); delapazalestrello@gmail.com (A.M.d.L.P.-E.); sergioperfer@gmail.com (S.P.-F.); vallergm@gmail.com (V.R.-M.); malemanv@ull.edu.es (R.A.-V.); 2 Gerencia de Atención Primaria de Tenerife, 38320 San Cristóbal de La Laguna, Spain; cristinagarciamarichal@gmail.com; 3 Departamento de Medicina Interna, Dermatología y Psiquiatría, Universidad de La Laguna, 38320 San Cristóbal de La Laguna, Spain; egonrey@ull.edu.es

**Keywords:** SARS-CoV-2, COVID-19, long term mortality, in-hospital mortality, risk factors

## Abstract

Background: The long-term survival of patients hospitalized with COVID-19 and the factors associated with poorer survival months after infection are not well understood. The aims of the present study were to analyze the overall mortality 10 months after admission. Methods: 762 patients with COVID-19 disease were included. Patients underwent a complete clinical evaluation, routine laboratory analysis and chest X-ray. Data collected included demographic and clinical data, such as vascular risk factors, tobacco or alcohol use, comorbidity, and institutionalization. Results: Ten-month mortality was 25.6%: 108 deaths occurred in-hospital, while 87 patients died after discharge. In-hospital mortality was independently related to NT-proBNP values > 503.5 pg/mL [OR = 4.67 (2.38–9.20)], urea > 37 mg/dL [3.21 (1.86–7.31)] and age older than 71 years [OR = 1.93 (1.05–3.54)]. NT-proBNP values > 503.5 pg/mL [OR = 5.00 (3.06–8.19)], urea > 37 mg/dL [3.51 (1.97–6.27)], cognitive impairment [OR = 1.96 (1.30–2.95), cancer [OR = 2.23 (1.36–3.68), and leukocytes > 6330/mm^3^ [OR = 1.64 (1.08–2.50)], were independently associated with long-term mortality. Conclusions: the risk of death remains high even months after COVID-19 infection. Overall mortality of COVID-19 patients during 10 months after hospital discharge is nearly as high as that observed during hospital admission. Comorbidities such as cancer or cognitive impairment, organ dysfunction and inflammatory reaction are independent prognostic markers of long-term mortality.

## 1. Introduction

SARS-CoV-2 infection has produced a global pandemic with enormous health and socio-economic consequences. SARS-CoV-2 is a member of the Orthocoronavirinae, closely related to SARS-CoV and MERS-CoV, both of which have also caused severe illness in humans [1]. Between January 2020 and December 2021, deaths directly related to SARS-CoV-2 infection in the world totalled 5.94 million [2].

Patients with SARS-CoV-2 infection may present with a variety of clinical manifestations, ranging from no symptoms to critical illness. In general, adults with SARS-CoV-2 infection can be classified into the following disease severity categories: (a) asymptomatic or pre-symptomatic infection: those individuals who do not have clinical COVID-19 but have tested positive for the virus. The percentage of individuals who present with asymptomatic infection and progress to clinical disease is unclear; (b) mild disease: patients presenting with mild symptoms. The most common symptoms are headache, sore throat, cough, fever, malaise, asthenia, myalgia, nausea, vomiting, diarrhea, anosmia and ageusia, without evidence of viral pneumonia or hypoxia. Patients aged ≥50 years, especially those aged ≥65 years, patients with certain underlying comorbidities, and patients who are immunosuppressed, unvaccinated, or not up to date with COVID-19 vaccinations are at higher risk of disease progression; (c) moderate disease: clinical signs of pneumonia but no signs of severe pneumonia, including SpO2 ≥ 90% on room air. Patients with moderate disease should be closely monitored; (d) severe disease: clinical signs of pneumonia plus one of the following: oxygen saturation of less than 90% on room air, severe respiratory distress or respiratory rate >30 breaths/min; (e) critical illness: individuals presenting with acute respiratory distress syndrome, sepsis, septic shock, acute thrombosis and/or multi-organ dysfunction [3]. The exact triggers for severe disease are also not known, but it appears that host factors are mainly involved in the pathogenesis [4].

Predictors of COVID-19 severity are closely linked to predictors of mortality. Several factors have been linked to severity and poor prognosis, such as advanced age [5], male sex [6], obesity [7], hypertension [8], cardiovascular disease [9], diabetes mellitus, [10], cancer [11], tobacco consumption [12] and chronic obstructive pulmonary disease [13]. Also, chronic liver disease and chronic kidney disease are associated with poor prognosis [14,15]. Clinical variables such as dyspnea [16,17] or hypoxia [4], radiographic features [4] and some biochemical parameters have also been related to mortality [18,19,20]. Zhang et al. found that elevated levels of C-reactive protein (CRP), lactate dehydrogenase (LDH), or the presence of lymphopenia were associated with a higher probability of admission to intensive care and worse prognosis [21].

The findings described above refer to short-term evolution. In contrast, there are not many studies that examine survival over extended periods. In addition, throughout the pandemic, attempts were made to describe the importance of comorbidity and frailty in the prognosis of COVID survivors. In a recent study, survival at 180 days was lower in those patients with lower previous cognitive and functional status [22], which underlines the importance of the existence of pre-existing illness or frailty. In addition, people who have suffered a COVID infection were at increased risk of death by any cause at 6 months, supporting the hypothesis that the risk of poor clinical outcome persists several months after discharge [23], mainly in elderly patients [24]. Several observations have pointed out that COVID-19 infection may alter immune response and trigger a smoldering inflammatory status that may be responsible for the development of the so-called post-COVID syndrome [25] and may aggravate the clinical course of co-existing chronic conditions, which results in a worse functional situation and a higher risk of poorer prognosis.

Based on these facts and considering that in our clinical setting many patients with COVID infection were also affected by other chronic conditions, the aims of the present study were to analyze overall mortality 10 months after admission for COVID-19, focusing on the influence of chronic diseases on prognosis. In-hospital mortality and factors related to in-hospital mortality were also analyzed as a secondary objective.

## 2. Materials and Methods

We prospectively included 762 patients (403 men), aged 68.4 ± 17.9, consecutively admitted to the COVID-19 Unit of our hospital due to COVID-19 disease from March 2020 to December 2020, the period covering the first three waves of the pandemic. Admission criteria were at least one of the following: (a) medical criteria: age over 60 years, high blood pressure and/or cardiovascular disease (include congestive heart failure and coronary artery disease), obesity (BMI > 30 Kg/m^2^), chronic lung disease, neoplasia, patients on immunosuppressive treatment, chronic renal disease, transplant recipients. (b) medical factors suggestive of higher risk of severe disease and/or decompensation: bilateral infiltrates on X-ray or rapidly progressive infiltrates (within 24–48 h), severe respiratory failure (tachypnoea > 30 respirations per minute (rpm), pulse oximetry saturation < 93%, PaO_2_/FiO_2_ < 300), D-dimer > 1000 ng/mL or/and rising, prolonged INR, CRP > 100 mg/L or rising, LDH > 245 U/L, troponin × 2 upper limit of normal (14 pg/mL), lymphopenia < 1000/mm^3^, plateletopenia < 100,000/mm^3^ or ferritin > 500 mg/dL or progressively increasing levels. (c) social/environmental factors: inability to care for self, resident of group facility or living with other high-risk people without an ability to isolate (no alternative living situation or only sharing spaces—bedroom/bathroom). Patients with a COVID-19 infection confirmed by real-time reverse transcription-polymerase chain reaction (RT-PCR) and with at least one admission criterion were included in this study. At admission, patients underwent complete clinical evaluation, routine laboratory analysis, and chest X-ray. The data collected included demographic and clinical data, such as vascular risk factors, toxic habits (active smoking, alcohol consumption or other drug use), comorbidity, and institutionalization. Clinical variables recorded were fever, dyspnea, oxygen saturation, cough, asthenia, diarrhea, headache, vomiting, and myalgia. We evaluated evidence of pneumonia on a chest X-ray, oxygen requirements and oxygen devices, the need for admission to an intensive care unit and the need for orotracheal intubation. Duration from onset of symptoms to hospital admission, length of stay in intensive care unit, and length of stay in hospital were also recorded.

Laboratory parameters included the assessment of a complete blood count, the biochemical profile (including liver and renal function, blood glucose, coagulation profile, lactate dehydrogenase and electrolytes), serum troponin, N-terminal pro-brain natriuretic peptide (NT-proBNP), and proinflammatory markers as procalcitonin, C-reactive protein (RCP) and serum ferritin. For each laboratory parameter, the median was determined as the cut-off point.

Frequency of examinations was determined by the treating physician. Patients were treated according to a local protocol that included corticosteroids (Dexamethasone 6 mg oral or intravenous for 10 days) if patient suffered respiratory insufficiency, antivirals (darunavir and remdesivir) and/or the IL-6 receptor antagonist Tocilizumab. Protocol included routine thromboprophylaxis depending on the risk of thrombosis, thrombocytopenia, and renal function. A total of 108 patients died during admission. After hospital discharge, 518 patients were followed up during 309 ± 173 days (median 311 (IQR 259–510 days): 203 patients who had suffered moderate to severe illness were seen as outpatients in a post-COVID unit, and 315 patients were followed up by their primary care physician. A total of 87 patients died during the follow-up period.

The study was conducted according to the guidelines of the Declaration of Helsinki and approved by the Institutional Ethics Committee of the Hospital Universitario de Canarias (code CHUC_2021_39). All patients gave verbal consent to be included in this study. Written consent was not collected during the pandemic’s peak to avoid paper contamination, in accordance with sanitary dispositions at that time, and the Institutional Ethics Committee granted an exemption from written informed consent.

### Statistical Analysis

A Kolmogorov–Smirnov test was used to explore if the variables showed a normal distribution or not. A Student’s *t*-test and a Mann–Whitney’s U test (for variables with non-parametric distribution) were used to compare mean values between two different groups. An χ2-test was used to compare qualitative variables. Survival was also analyzed using Kaplan Meier curves at both discharge and 10 months. We performed a Cox regression analysis to test the independence or not of the relationships between mortality and several variables. We applied the receiver operating characteristic (ROC) curves to calculate the area under the curve, of those variables with an independent prognostic value. We also performed interaction analyses. These analyses were performed with SPSS software (25.0) (Chicago, IL, USA).

## 3. Results

We included 762 patients (403 men, 53%), aged 68.4 ± 17.9 years. In relation to disease severity, 98 patients (12.9%) were asymptomatic, 85 (11%) had mild disease, while a larger proportion, totaling 355 individuals (46.6%), fell into the category of moderate disease. Moreover, 115 (15.1%) patients met the criteria for severe disease and 110 (14.4%) had critical disease. Overall, 10-month mortality was 25.6%: 108 deaths occurred in-hospital (55.4% of the overall 10-month mortality), while 87 patients died after discharge (44.6% of the overall 10-month mortality). Cardiovascular diseases were detected in 261 (35%) patients, especially coronary artery disease (8.8%). Other comorbidities were pulmonary diseases (162 patients, 20%), a central nervous system disorder (21.4% of patients had dementia), or neoplasms (12.3%). A total of 187 (25.6%) patients were institutionalized. The demographic and clinical characteristics of patients are shown in Table 1.

Clinical and biochemical variables are shown in Table 2, together with data related to ventilatory support, which were collected in 683 patients. Most patients (81.3%) required conventional oxygen therapy. Continuous positive airway pressure (CPAP) or noninvasive mechanical ventilation were needed in 69 (10.1%) patients and 59 (8.6%) underwent endotracheal intubation and invasive mechanical ventilation. A total of 116 patients (15.2%) required admission to intensive care units (ICUs). During admission, we identified 14 episodes of thrombosis (seven cases of pulmonary thromboembolism), and 70 (9.2%) patients developed a nosocomial infection.

### 3.1. In-Hospital Mortality

**Figure 1:**
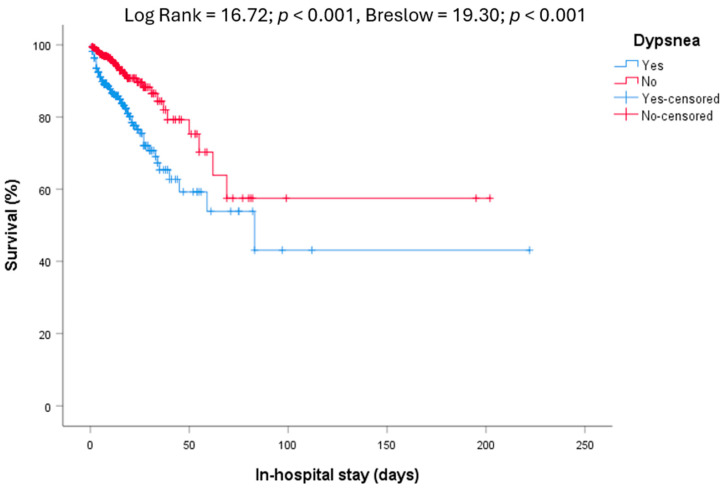
In-hospital mortality. Relationship between survival and dyspnea at admission (Log Rank = 16.72; *p* < 0.001, Breslow = 19.30; *p* < 0.001).

During admission, 108 patients died. Some clinical features are shown in Table 2 and univariate analysis are shown in Table S1 (Supplementary Materials). The age of non-survivors was higher (79.7 ± 13.5 vs. 66.5 ± 17.8 years; t = 8.92; *p* < 0.001). In the univariate analysis performed with the Kaplan–Meier method, we found higher mortality among patients who met the criteria for metabolic syndrome (Log Rank = 4.33; *p* = 0.037, Breslow = 4.07; *p* = 0.044), hypertensive heart disease (Log Rank = 16.93; *p* < 0.001, Breslow = 12.19; *p* < 0.001), congestive heart failure (Log Rank = 13.02; *p* < 0.001, Breslow = 9.85; *p* = 0.002), atrial fibrillation (Log Rank = 2.72; NS, Breslow = 4.18; *p* = 0.041), stroke (Log Rank = 1.68; NS, Breslow = 4.53; *p* = 0.033), dementia (Log Rank = 23.99; *p* < 0.001, Breslow = 22.87; *p* < 0.001), neoplasms (Log Rank = 12.97; *p* < 0.001, Breslow = 5.43; *p* = 0.020) and among patients who lived in nursing homes (Log Rank = 8.13; *p* = 0.004, Breslow = 12.66; *p* < 0.001). Symptomatic patients had a higher mortality (Log Rank = 8.35; *p* = 0.004, Breslow = 6.44; *p* = 0.011), especially those who reported dyspnea on admission (Log Rank = 16.72; *p* < 0.001, Breslow = 19.30; *p* < 0.001, Figure 1).

**Figure 2:**
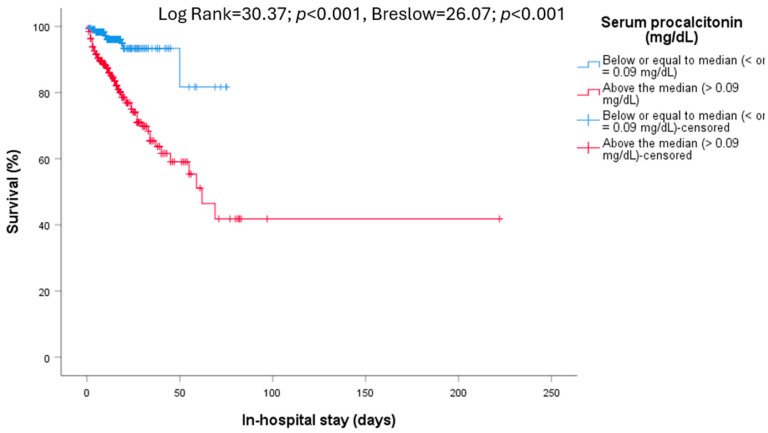
In-hospital mortality. Relationship between survival and serum procalcitonin levels (Log Rank = 30.37; *p* < 0.001, Breslow = 26.07; *p* < 0.001).

Stratifying the analytical parameters according to medians, we found that lower oxygen saturation on admission was associated with higher mortality (Log Rank = 27.54; *p* < 0.001, Breslow = 31.66; *p* < 0.001) in addition to lymphopenia (Log Rank = 9.50; *p* = 0.002, Breslow = 15.60; *p* < 0.001), leukocytosis (Log Rank = 13.45; *p* < 0.001, Breslow = 14.97; *p* < 0.001), and those who had higher values of urea (Log Rank = 39.98; *p* < 0.001, Breslow = 42.93; *p* < 0.001), serum creatinine (Log Rank = 29.42; *p* < 0.001, Breslow = 20.86; *p* < 0.001), C-reactive protein (Log Rank = 10.70; *p* = 0.001, Breslow = 13.67; *p* < 0.001), procalcitonin (Log Rank = 30.37; *p* < 0.001, Breslow = 26.07; *p* < 0.001, Figure 2), lactate dehydrogenase (Log Rank = 17.64; *p* < 0.001, Breslow = 28.65; *p* < 0.001), ferritin (Log Rank = 9.44; *p* = 0.002, Breslow = 11.47; *p* = 0.001), and D-dimer (Log Rank = 11.51; *p* = 0.002, Breslow = 18.21; *p* < 0.001).

Values above the median of troponin and natriuretic peptide were also associated with increased mortality (*p* < 0.001 in both cases) as were elevated levels of serum interleukin 6 (IL-6) (Log Rank = 4.02; *p* = 0.045, Breslow = 7.01; *p* = 0.008). Patients who required noninvasive mechanical ventilation had a poorer prognosis than those with other ventilatory support (Log Rank = 8.03; *p* = 0.045, Breslow = 10.64; *p* = 0.014). In contrast, patients requiring admission to critical care units had lower in-hospital mortality (Log Rank = 4.21; *p* = 0.040, Breslow = 4.76; *p* = 0.029). We failed to find any relationship between in-hospital mortality and treatments used.

We performed a multivariate Cox regression analysis to identify independent predictors of in-hospital mortality, and included those variables which were statistically significant in the univariate analysis (Table S1). The results from the multivariate Cox regression were values above the median of natriuretic peptide [HR = 4.67 (2.38–9.20)], urea [HR = 3.21 (1.86–7.31)], and age [HR = 1.93 (1.05–3.54), and were independently related to mortality.

### 3.2. Long-Term Mortality

A total of 195 patients died. Most patients died as a direct consequence of sepsis (34.5%). Ictus or cardiovascular diseases were reported in eight patients (9.2%) and advanced cancer was also a leading cause of death (five patients, 5.7%). A high proportion of deaths occurred in nursing homes (28.7%), in all of which the causes were reported as cardiorespiratory arrest. In 18 cases, information about the cause of death was unavailable.

Some clinical features are shown in Table 2. The age of non-survivors was higher (79.4 ± 13.9 vs. 64.6 ± 17.5 years; t = 12.00; *p* < 0.001). We found higher mortality in patients with hypertension (Log Rank = 16.19; *p* < 0.001, Breslow = 14.76; *p* < 0.001), dyslipidemia (Log Rank = 4.95; *p* = 0.026, Breslow = 4.08; *p* = 0.043) and metabolic syndrome (Log Rank = 14.37; *p* < 0.001, Breslow = 13.47, *p* < 0.001). No relationships were observed between diabetes mellitus, obesity or hyperuricemia and mortality.

**Figure 3:**
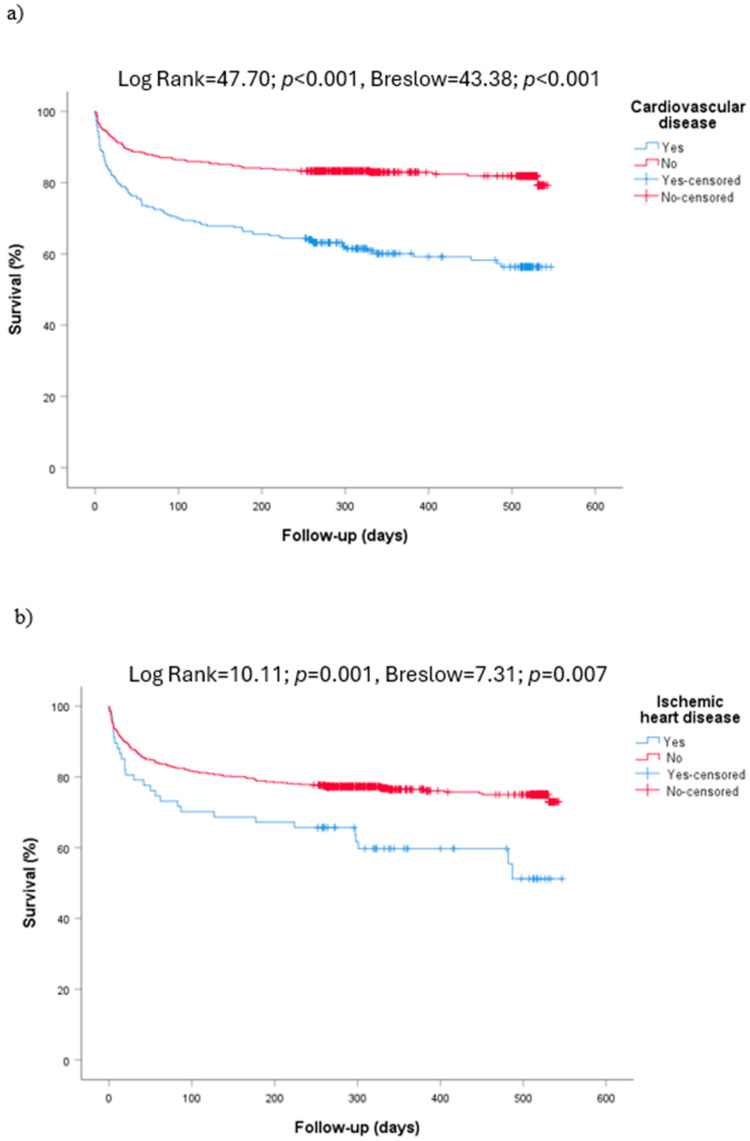

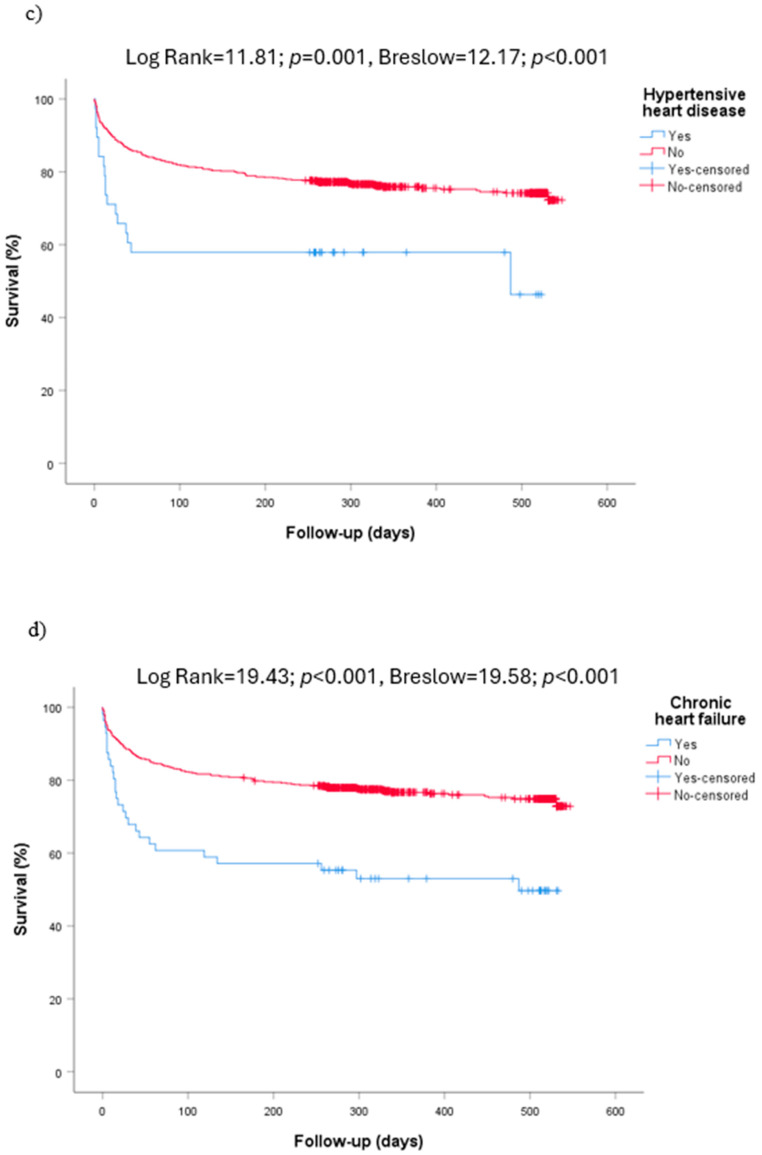
Overall mortality. (**a**) Relationship between survival and cardiovascular diseases. (**b**) Relationship between survival and ischemic heart disease. (**c**) Relationship between survival and hypertensive heart disease. (**d**) Relationship between survival and chronic heart failure.

Cardiovascular diseases were related to mortality (Log Rank = 47.70; *p* < 0.001, Breslow = 43.38; *p* < 0.001), even when analyzed separately (Figure 3a–d).

Patients with chronic obstructive pulmonary disease died more frequently (Log Rank = 9.20; *p* = 0.002, Breslow = 6.84; *p* = 0.009), as did patients with cognitive impairment (Log Rank = 82.95; *p* < 0.001, Breslow = 77.16; *p* < 0.001), Parkinson’s disease (Log Rank = 4.39; *p* = 0.036, Breslow = 4.81; *p* = 0.028), and neoplasms (Log Rank = 21.70; *p* < 0.001, Breslow = 20.57; *p* < 0.001) and patients who lived in nursing homes (Log Rank = 42.41; *p* < 0.001, Breslow = 40.56; *p* < 0.001).

Patients who were symptomatic on admission had no increased long-term mortality compared to asymptomatic patients except for those with dyspnea (Log Rank = 8.89; *p* = 0.003, Breslow = 11.99; *p* = 0.001). Stratifying the analytical parameters according to the median, we found that lower oxygen saturation on admission was associated with higher long-term mortality (Log Rank = 12.19; *p* < 0.001, Breslow = 17.82; *p* < 0.001) in addition to lymphopenia (Log Rank = 8.50; *p* = 0.004, Breslow = 13.05; *p* < 0.001), leukocytosis (Log Rank = 20.99; *p* < 0.001, Breslow = 23.48; *p* < 0.001), and those who had higher values of urea (Log Rank = 107.14; *p* < 0.001, Breslow = 105.40; *p* < 0.001), serum creatinine (Log Rank = 28.51; *p* < 0.001, Breslow = 27.25; *p* < 0.001), C-reactive protein (Log Rank = 12.57; *p* < 0.001, Breslow = 14.99; *p* < 0.001), procalcitonin (Log Rank = 30.34; *p* < 0.001, Breslow = 36.42; *p* < 0.001), lactate dehydrogenase (Log Rank = 6.52; *p* = 0.011, Breslow = 11.74; *p* = 0.001), and D-dimer (Log Rank = 38.84; *p* < 0.001, Breslow = 38.22; *p* < 0.001). Values above the median of troponin (Log Rank = 37.50; *p* < 0.001, Breslow = 35.24; *p* < 0.001) and natriuretic peptide (Log Rank = 53.74; *p* < 0.001, Breslow = 51.59; *p* < 0.001) were also associated with increased mortality, as was also observed in relation to Il-6 levels over the median (Log Rank = 8.38; *p* = 0.004, Breslow = 8.86; *p* = 0.003).

No statistically significant relationship was found between admission to ICUs and overall mortality. Patients treated with remdesivir showed a trend toward improved survival (Log Rank = 3.84; *p* = 0.050, Breslow = 3.74; *p* = 0.053). No relationships were found between mortality and other treatments used.

We performed a multivariate Cox regression analysis to identify independent predictors of in-hospital mortality, and included those variables which were statistically significant in the univariate analysis (Table S1). Values above the median of natriuretic peptide [HR = 5.00 (3.06–8.19)], urea [HR = 3.51(1.97–6.27)], cognitive impairment [HR = 1.96 (1.30–2.95)], cancer [HR = 2.23 (1.36–3.68)], and leukocytosis [HR = 1.64 (1.08–2.50)] were independently related to mortality.

We analyzed the interaction effect of cancer in two Cox models: model 1: cancer + age + cancer*age, and model 2: cancer + sex + cancer*sex. No significant effects of interaction were found for cancer*age (*p* = 0.860) and cancer*sex (*p* = 0.976). We also analyzed the interaction effect of cognitive impairment in two Cox models: model 1: cognitive impairment + age + cognitive impairment*age, and model 2 cognitive impairment + sex + cognitive impairment*sex. No significant effects of interaction were found for cognitive impairment*age (*p* = 0.555) and cognitive impairment*sex (*p* = 0.055).

**Figure 4:**
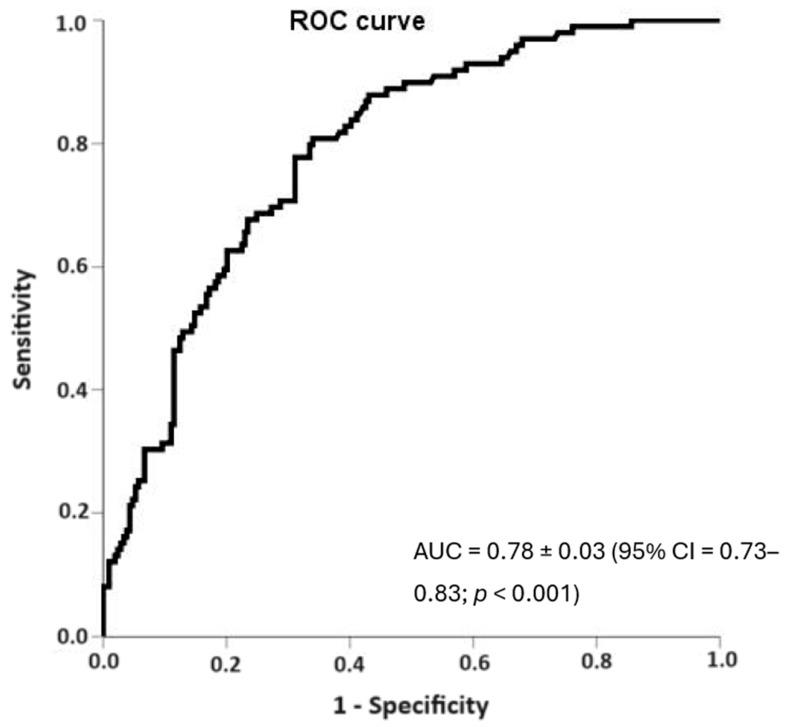
Relationship between NT-proBNP values and overall mortality [AUC = 0.78 ± 0.03 (95% CI = 0.73–0.83; *p* < 0.001)].

The relationship between NT-proBNP values and overall mortality was also evident when a ROC curve was performed in order to analyze the sensitivity and specificity of NT-proBNP, with an AUC of 0.78 ± 0.03 (95% CI = 0.73–0.83; *p* < 0.001, Figure 4). Similar findings were obtained with urea (AUC = 0.76 ± 0.02 (95% CI = 0.74–0.81; *p* < 0.001) and leukocytosis (AUC = 0.63 ± 0.24 (95% CI = 0.58–0.68; *p* < 0.001).

## 4. Discussion

The present study describes the clinical and epidemiological characteristics of a large case series of patients with COVID infection in order to analyze the overall mortality 10 months after admission for COVID-19, with a focus on the influence of chronic diseases on prognosis. In-hospital mortality and related factors were also analyzed.

A total of 108 patients died during admission (14.2%). The mortality rate is similar to that described in other hospitals in our country [26] but lower than that reported by the SEMI-COVID-19 registry, which was 21% [27]. However, other authors describe significantly higher mortality in the earlier stages of the pandemic [28]. Survival improved as the pandemic progressed, which may suggest improvements in clinical care [29]. Additionally, a recent meta-analysis showed a mortality rate of 16% for general COVID-19 patients admitted to hospital [30]. Age, comorbidities such as cardiovascular disease or chronic kidney disease, as well as other medical factors suggesting risk of a poor clinical outcome were considered when deciding on admission to our hospital. The mean age of the patients was 68.4 ± 17.9 years and up to 25.6% of the patients lived in nursing homes, so that the patients included in this study are presumably frailer and with worse functional status than the general population.

As mentioned in the introduction, predictors of COVID-19 severity are closely linked to predictors of mortality. Thus, risk factors such as age or comorbidity were closely related to mortality. We found that in-hospital mortality is influenced by age, comorbidity, some symptoms, organ dysfunction, inflammatory reaction, and the need for non-invasive mechanical ventilation. Our results are consistent with those of a meta-analysis by Shi et al., who reported that the increased fatality rate included older age, male sex, current smoking, baseline comorbidities (especially chronic kidney, respiratory, and cardio-cerebrovascular diseases), symptoms of dyspnea, complications during hospitalization, corticosteroid therapy, and a severe condition [17].

Thus, when performing a multivariate analysis, we found that levels above 504.50 pg/mL of NT-proBNP and 37 mg/dL of urea and age above the median (71 years), in that order, are independent prognostic markers of in-hospital mortality. These findings reinforce the previously described evidence that cardiac [31,32] or renal dysfunction [33,34] are prognostic markers, as well as age [35]. Also, our results highlight the prognostic value of NT-proBNP, a marker typically used to assess cardiac function. Elevated NT-proBNP levels in COVID-19 patients were indicative not only of heart strain but also of a broader systemic impact, reflecting the multi-organ involvement often seen in severe cases.

A key aspect of this work is that patients were followed-up after discharge during a mean period of 10 months. Few studies have included such long observational periods [36,37,38]. During the follow-up period, 87 patients died (13.3% of the 654 who lived after discharge). This proportion is substantially higher than that described in other studies, although the published literature on long-term follow-up remains scarce. Novelli et al. found a 1-year post-discharge mortality rate of 3.7%, although 1-year mortality was 33.6% (also including patients who died during admission), a figure higher than that reported in our study (25.6%) [36]. Other authors report all-cause mortality rates of hospitalized COVID-19 patients during 1 year after discharge ranging from 4.7% to 9.3% [22,39,40] although higher mortality rates have been reported in the literature [41,42]. Our results are similar to those reported by Li et al. who found a mortality of 11.2% in hospitalized patients with COVID-19 after a mean follow-up of 72.8 days [43] and with those of Bertolotti et al., who described a post-discharge mortality of 16.2% and an overall mortality of 38.7% [44] after a 6-month outpatient follow-up period. In addition, the follow-up time was longer in our study, which could lead to higher mortality. Contrary to the results reported by Novelli et al., our study suggests that COVID-19 mortality remained high during the months following discharge [36].

Variables related to overall mortality were NT-proBNP (>504.50 pg/mL), urea (>37 mg/dL), the presence of cognitive impairment, neoplasms, and total leukocyte count above the median (6330/mm^3^), in that order. Recently, a NT-proBNP cut-off level of 1022.50 pg/mL has been identified as an independent predictor of 1-year mortality in COVID-19 patients [45]. We have found that even lower values of NT-proBNP (>504.50 pg/mL) can predict long-term mortality. Impaired renal function and white blood cell count have also been related as independent predictors of long-term mortality. Lucijanić et al. found that both urea on admission >10.5 mmol/L and white blood cell count >7 × 10^9^/L were independently related to mortality [46]. Also, patients with end-stage renal disease have significantly higher odds of all-cause 1-year mortality [37]. The identification of these biomarkers not only aids in prognostication but also opens avenues for potential therapeutic interventions. For instance, closer monitoring and early intervention in patients with elevated NT-proBNP or urea levels might improve outcomes. Additionally, these findings could help in stratifying patients for more intensive follow-up and personalized care plans post-discharge.

Predictors of post-discharge mortality also include pre-existing comorbidities such as cognitive impairment or malignancy. Dementia enhanced the severity and mortality risk of COVID-19 [47], leading to an increased mortality risk of more than threefold during the 6 months after admission [22]. Cancer was related to increased mortality risk that persisted until the end of the first year after infection [38] and may increase the risk of mortality by up 3.64-fold [36]. In relation to this finding, it is important to note that the pandemic probably caused delays in the diagnosis of neoplasms, which has had adverse consequences for the initiation of treatment, mortality rates, and years of life lost. A recently published study with a 3-year follow-up of patients with COVID infection who required hospital admission during the infection describes that the main causes of death after hospital discharge were heart disease, influenza, sepsis, COVID itself, and acute respiratory distress syndrome. As an important limitation, this work only included patients infected during the first wave of the pandemic [48]. These results are consistent with those of our study, as during the follow-up period most patients died as a direct consequence of sepsis (34.5%) and stroke or cardiovascular diseases were reported in eight patients as the cause of death (9.2%).

Walle-Hansen et al. conducted a study of patients with COVID infection over 60 years of age who were admitted to hospital. The 6-month mortality rate was 21% overall, and analyzed by age group, 36% of those who died were aged 75 years or older and 8% of those who died were aged 60–74 years [49]. Frail older adults have a pre-existing immunopathological basis that exposes them to an increased risk of poor outcomes and mortality from COVID-19 and poor response to COVID-19 vaccination [50]. It is noteworthy that in our study, age is not a prognostic marker. We consider that this finding should be interpreted with caution, given that age does influence both the clinical course and the in-hospital mortality rate. Although perhaps the increased severity of COVID-19 infection in the elderly may explain that most will die during admission, more follow-up studies are needed to be able to draw firm conclusions regarding age and long-term mortality.

This study has some limitations. Because of the study design, including patients admitted to a single centre during the first three waves of the pandemic, some data were not collected, as shown in Table 1 and Table 2. Also, in 18 cases information about death was unavailable. However, our study evaluates predictors of in-hospital mortality and post-discharge mortality, and the multivariate analysis is not affected by the cause of death. In addition, we did not include vaccinated patients because the clinical evolution of COVID-19 patients is different in the post-vaccination period, and we believe that the clinical course of vaccinated patients is not comparable with that of the non-vaccinated ones. We acknowledge the limitation that we did not include a healthy control group. Also, the study design does not allow the establishment of causal links between COVID and mortality. However, our purpose was to study mortality in patients with COVID-19 disease and how it relates to comorbidities and disease characteristics. The strengths of the study are the extensive collection of both clinical and analytical variables and the follow-up period. Knowing how patients who have suffered a COVID infection evolve can help us to understand the behavior of this disease and to try to design strategies to improve patient survival such as, for example, enhancing telemedicine surveillance.

## 5. Conclusions

Overall mortality of COVID-19 patients during 10 months after hospital discharge is nearly as high as that observed during hospital admission. Comorbidities such as cancer or cognitive impairment, organ dysfunction and inflammatory reaction are independent prognostic markers of long-term mortality. These findings may help identify patients where more active surveillance should be performed after discharge in order to improve long-term prognosis.

## Figures and Tables

**Table 1:** Demographic and comorbidities of patients, in relation to in-hospital and long-term mortality.

		In-Hospital Mortality			Long-Term Mortality		
	All (762)	Survivors (654)	Dead (108)	*p*-Value	Survivors (567)	Dead (195)	*p*-Value
Age (mean ± SD) >71; n (%)	68.4 ± 17.9 376 (49.3)	79.7 ± 13.0 290 (44.3)	66.5 ± 17.8 86 (79.6)	<0.001	64.6 ± 17.5 220 (38.8)	79.4 ± 13.9 156 (80.0)	<0.001
Sex (M/F); n (%)	403/359 (52.9/47.1)	349/305 (53.4/46.6)	50/50 (54/54)	NS	52.4/47.6 (297/270)	54.4/45.6 (106/89)	NS
Tobacco use (746)				NS			NS
- Current smokers	71 (9.3)	67 (10.2)	4 (3.7)		57 (10.0)	14 (7.2)	
- Former smokers	182 (23.9)	152 (23.2)	30 (27.8)		132 (23.3)	50 (25.6)	
- Non-smokers	493 (64.7)	419 (64.1)	74 (68.5)		366 (64.6)	127 (65.1)	
Alcohol consumption (746) *				NS			NS
- Hazardous alcohol consumption	79 (10.4)	72 (11.0)	7 (6.5)		60 (10.6)	19 (9.7)	
- Former drinkers	32 (4.2)	27 (4.1)	5 (4.6)		23 (4.1)	9 (4.6)	
- Non-drinkers	635 (83.3)	539 (82.4)	96 (88.9)		472 (83.2)	163 (83.6	
Metabolic syndrome (746)	384 (51.5)	312 (48.9)	72 (66.7)	<0.001	(265) 47.7	(119) 62.3	<0.001
Hypertension (746)	428 (57.4)	352 (55.2)	76 (70.4)	NS	295 (53.2)	133 (69.6)	<0.001
Dyslipidemia (746)	365 (48.9)	304 (47.6)	61 (56.5)	NS	258 (46.5)	107 (56.0)	0.026
Diabetes mellitus (746)	246 (33.0)	203 (31.8)	43 (39.8)	NS	176 (31.7)	70 (36.6)	NS
Obesity (746)	191 (25.6)	159 (24.9)	32 (29.6)	NS	139 (25.0)	52 (27.2)	NS
Cardiovascular diseases (746)	261 (35.0)	204 (32.0)	57 (52.8)	NS	155 (27.9)	106 (55.5)	<0.001
- Coronary artery disease (745)	66 (9.0)	51 (8.0)	16 (14.8)	NS	39 (7.0)	28 (14.7)	0.001
- Congestive heart failure (746)	56 (7.5)	38 (6.0)	18 (16.7)	<0.001	29 (5.2)	27 (14.1)	<0.001
- Hypertensive heart disease (746)	38 (5.1)	25 (3.9)	13 (12.0)	<0.001	21 (3.8)	17 (8.9)	0.001
- Atrial fibrillation (744)	81 (10.9)	63 (9.9)	18 (16.7)	NS	43 (7.8)	38 (19.9)	<0.001
Stroke (746)	42 (5.6)	33 (5.2)	9 (8.3)	NS	24 (4.3)	18 (9.4)	0.004
COPD (746)	59 (7.9)	48 (7.5)	11 (10.2)	NS	34 (6.1)	25 (13.1)	0.002
Asthma (746)	55 (7.4)	47 (7.4)	8 (7.4)	NS	44 (7.9)	11 (5.8)	NS
Dementia (746)	163 (21.8)	122 (19.1)	41 (38.0)	<0.001	79 (14.2)	84 (44.0)	<0.001
Parkinson’s disease (751)	19 (2.5)	15 (2.3)	4 (3.7)	NS	11 (2.0)	8 (4.2)	0.036
Neoplasm (745)	94 (12.6)	67 (10.5)	27 (25.0)	<0.001	52 (9.4)	42 (22.0)	<0.001
Chronic kidney disease	50 (6.6)	32 (4.9)	18 (14.2)	<0.001	26 (4.6)	24 (12.3)	<0.001
Institutionalized (731)	187 (25.6)	151 (24.2)	36 (33.6)	0.004	106 (19.7)	81 (42.2)	<0.001

NS: no statistical significance. * Risk dose of alcohol consumption: >20 g/day in men and >10 g/day in women.

**Table 2:** Ventilatory support, clinical and biochemical variables in relation to in-hospital and long-term mortality.

		In-Hospital Mortality			Long-Term Mortality		
	All (762)	Survivors (654)	Dead (108)	*p*-Value	Survivors (567)	Dead (195)	*p*-Value
Oxygen saturation <94% (724); n (%)	399 (55.1)	313 (50.6)	86 (81.9)	<0.001	277 (51.5)	122 (65.6)	<0.001
Oxygen support (683)				NS			NS
Conventional	555 (81.3)	479 (83.2)	76 (71.0)		398 (80.6)	157 (83.1)	
CPAP/NIVM	69 (10.1)	54 (9.4)	15 (14.0)		54 (10.9)	15 (7.9)	
Invasive MV	59 (8.6)	43 (7.5)	16 (15.0)		42 (8.5)	17 (9.0)	
Critical Care	116 (15.2)	95 (14.5)	21 (19.4)	0.040	94 (16.6)	22 (11.3)	NS
Symptomatic	664 (87.1)	561 (85.8)	103 (95.4)	<0.001	494 (87.1)	170 (7.2)	NS
Severity of illness				<0.001			<0.001
- Asymptomatic	98 (12.9)	93 (14.2)	5 (4.6)		73 (12.9)	25 (12.8)	
- Mild disease	85 (11.0)	82 (12.5)	3 (2.8)		62 (10.9)	23 (11.8)	
- Moderate disease	355 (46.6)	317 (48.5)	38 (35.2)		280 (49.3)	75 (38.5)	
- Severe disease	115 (15.1)	74 (11.2)	41 (38.0)		67 (11.8)	48 (24.6)	
- Critical disease	110 (14.4)	89 (13.6)	21 (19.4)		86 (15.1)	24 (12.3)	
Fever	423 (55.5)	360 (55.0)	63 (58.3)	NS	331 (58.4)	92 (47.2)	0.015
Cough	364 (47.8)	324 (49.5)	40 (37.0)	NS	301 (53.1)	63 (32.3)	<0.001
Dyspnea	390 (51.2)	315 (48.2)	75 (69.4)	<0.001	273 (48.1)	117 (60.0)	0.003
Asthenia	262 (34.4)	236 (36.1)	26 (24.1)	NS	213 (37.6)	49 (25.1)	0.003
Diarrhea	132 (17.3)	121 (18.5)	11 (10.2)	NS	113 (19.9)	19 (9.7)	0.001
Leukocytes >6330/mm^3^ (733)	367 (50.1)	333 (53.1)	34 (32.1)	<0.001	300 (55.1)	67 (35.4)	<0.001
Lymphocytes <1000/mm^3^ (733)	368 (50.2)	292 (46.6)	76 (71.7)	<0.001	258 (47.4)	110 (58.2)	0.004
Creatinine >0.87 mg/dL (735)	368 (50.1)	285 (45.5)	83 (76.9)	<0.001	241 (44.3)	127 (66.5)	0.001
Urea >37 mg/dL (735)	368 (50.1)	276 (44.0)	92 (85.2)	<0.001	212 (39.0)	156 (81.7)	0.001
C-reactive protein >48 mg/dL (731)	366 (50.1)	289 (46.3)	77 (72.0)	<0.001	251 (46.4)	115 (60.5)	0.001
Procalcitonin >0.09 mg/dL (631)	326 (51.7)	253 (46.3)	73 (85.9)	<0.001	217 (46.0)	109 (68.6)	<0.001
LDH >270 UI/L (637)	319 (50.1)	252 (46.0)	67 (75.3)	<0.001	229 (47.8)	90 (57.0)	0.011
Ferritin >402 mg/dL (687)	344 (50.1)	280 (47.1)	64 (69.6)	0.002	248 (48.2)	96 (55.8)	NS
D-dimer >855 mg/dL (675)	338 (50.1)	270 (46.3)	68 (73.9)	0.002	218 (43.2)	120 (70.6)	<0.001
Troponin >15.2 pg/mL (183)	92 (50.3)	63 (42.0)	29 (87.9)	<0.001	49 (36.3)	43 (89.6)	<0.001
Natriuretic peptide >503.5 pg/mL (308)	154 (50.0)	99 (40.7)	55 (84.6)	<0.001	74 (35.4)	80 (80.8)	<0.001
IL-6 >26.6 pg/mL (162)	81 (50.0)	60 (43.8)	21 (84.0)	0.045	58 (44.6)	23 (71.9)	0.004

NS: no statistical significance.

## Data Availability

The data presented in this study are available on request from the corresponding author.

## Supplementary Materials

The following supporting information can be downloaded at: https://www.mdpi.com/article/10.3390/diseases12060123/s1, Table S1: Univariate analysis.
