# Supplementary material for: Mortality and COVID Infection: Predictors of Mortality 10 Months after Discharge
Source: Diseases. 2024 Jun 5;12(6):123. doi: 10.3390/diseases12060123 (PMC11203287; doi:10.3390/diseases12060123)
Supplement: Supplementary file 1 [file diseases-12-00123-s001.zip › diseases-3030753-supplementary.pdf]

Supplementary materials.

Table S1: Univariate analysis.

	In-hospital mortality			Long-term mortality	
	Beta Coef. (95%), p			Beta Coef. (95%), p	
Age (years)	1.06 (1.04-1.07)	0.000		1.06 (1.05-1.07)	0.000
Men	0.80 (0.55-1.17)	0.247		1.45 (0.79-1.39)	0.757
Metabolic syndrome	1.53 (1.02-2.28)	0.039		1.73 (1.29-2.32)	0.000
Hypertension	1.49 (0.99-2.26)	0.058		1.84 (1.35-2.50)	0.000
Dislipidemia	1.22 (0.83-1.78)	0.311		1.39 (1.05-1.85)	0.023
Diabetes mellitus	1.16 (0.79-1.71)	0.456		1.24 (0.93-1.67)	0.146
Obesity	1.13 (0.75-1.71)	0.562		1.13 (0.83-1.56)	0.438
Cardiovascular diseases:	1.89 (1.29-2.76)	0.001		2.60 (1.96-3.46)	0.000
Coronary artery disease	1.29 (0.75-2.20)	0.358		1.89 (1.27-2.83)	0.002
Congestive heart failure	2.46 (1.48-4.09)	0.001		2.32 (1.53-3.51)	0.000
Hypertensive heart disease	3.17 (1.77-5.68)	0.000		2.33 (1.42-3.84)	0.001
Atrial fibrillation	1.53 (0.92-2.54)	0.103		2.22 (1.55-3.18)	0.000
Stroke	1.56 (0.79-3.10)	0.200		2.00 81.23-3.26)	0.005
COPD	1.17 (0.62-2.18)	0.629		1.88 (1.24-2.87)	0.003
Asthma	1.19 (0.58-2.44)	0.645		0.73 (0.40-1.33)	0.302
Dementia	2.59 (1.74-3.85)	0.000		3.54 (2.66-4.12)	0.000
Parkinson disease	1.198 (0.73-5.41)	0.181		2.08 (1.02-4.23)	0.043
Neoplasm	2.18 (1.41-3.38)	0.000		2.21 (1.56-3.12)	0.000
Chronic kidney disease	0.98 (0.55-1.73)	0.936		0.84 (0.31-2.27)	0.730
Institutionalized	1.80 (1.19-2.71)	0.005		2.55 (1.91-3.40)	0.000
Oxygen saturation < 94%	3.47 (2.11-5.71)	0.000		1.71 (1.27-2.32)	0.000
Symptomatic	3.45 (1.41-8.48)	0.007		1.03 (0.68-1.57)	0.896
Fever	1.15 (0.78-1.68)	0.478		0.71 (0.54-0.95)	0.019
Cough	0.73 80.49-1.08)	0.110		0.46 (0.34-0.62)	0.000
Dyspnea	2.29 (1.52-3.45)	0.000		1.55 (1.17-2.07)	0.003
Asthenia	0.64 (0.41-1.00)	0.048		0.62 (0.45-0.86)	0.004
Diarrhea	0.57 (0.30-1.06)	0.073		0.47 (0.29-0.75)	0.002
Critical Care	0.60 (0.36-0.98)	0.043		0.71 (0.46-1.01)	0.125
Leukocytes (x10e3/mL)	1.00 (1.00-1.00)	0.000		1.00 (1.00-1.00)	0.000
Lymphocytes (x10e3/mL)	1.00 (1.00-1.00)	0.000		1.00 (1.00-1.00)	0.008
Creatinine (mg/dL)	1.46 (1.29-1.65)	0.000		1.49 (1.34-1.65)	0.000
Urea (mg/dL)	1.01 (1.01-1.02)	0.000		1.02 (1.01-1.02)	0.000
C-reactive protein (mg/dL)	1.00 (1.00-1.00)	0.001		1.00 (1.00-1.00)	0.053
Procalcitonin (mg/dL)	1.02 (0.98-1.05)	0.305		1.00 (0.97-1.04)	0.770
LDH (U/L)	1.00 (1.00-1.00)	0.000		1.00 (1.00-1.00)	0.000
Ferritin (mg/dL)	1.00 (1.00-1.00)	0.001		1.00 (1.00-1.00)	0.001
D-dimer (mg/dL)	1.00 (1.00-1.00)	0.020		1.00 (1.00-1.00)	0.000
Troponin (pg/mL)	1.00 (1.00-1.00)	0.069		1.00 (1.00-1.00)	0.000
Natriuretic peptide (pg/mL)	1.00 (1.00-1.00)	0.002		1.00 (1.00-1.00)	0.000
IL-6 (pg/mL)	1.00 (1.00-1.00)	0.939		1.00 (1.00-1.00)	0.128
IL-6 (>26.6 pg/mL)	2.93 (0.98-8.78)	0.055		3.07 (1.43-6.61)	0.004
